# Dexketoprofen enhances NLRP3 activation via ATPase activity after canonical stimuli

**DOI:** 10.1007/s10787-025-01928-2

**Published:** 2025-09-23

**Authors:** Daniel Boy-Ruiz, Juan Miguel Suarez-Rivero, Inés Muela-Zarzuela, Mario D. Cordero

**Affiliations:** Department of Molecular Biology and Biochemical Engineering, Universidad Pablo de Olavide, 41013 Seville, Spain

**Keywords:** Inflammasome, Dexketoprofen, NLRP3, MCC950, ATPase

## Abstract

Inflammasomes are crucial elements of the innate immune system, responsible for triggering inflammation through the activation of caspase-1. Among them, the NLRP3 inflammasome plays a central role in various inflammatory and immune-related disorders. Dexketoprofen (DXK) is a widely used nonsteroidal anti-inflammatory drug (NSAID), although it has not been tested for its effects on the NLRP3 inflammasome pathway. In this study, we explored the influence of DXK on NLRP3 activation and its associated inflammatory responses in human macrophages. Our results showed that even at low concentrations, DXK enhances the release of IL-1β and promotes cell death upon stimulation with LPS and ATP, nigericin and LPS and nigericin, indicating it increases inflammasome activation. Docking and ATPase assays revealed that DXK binds to the NLRP3 NATCH domain, facilitating ATP hydrolysis and NLRP3 activation. Furthermore, treatment with DXK in combination with nigericin further increased IL-1β secretion, while the NLRP3 inhibitor MCC950 reduced the effects of DXK. These findings suggest that DXK amplifies inflammation in macrophages via NLRP3 activation, emphasizing the importance of caution in prolonged use, especially in autoinflammatory diseases where inflammasomes play a significant role.

**Graphical abstract:**

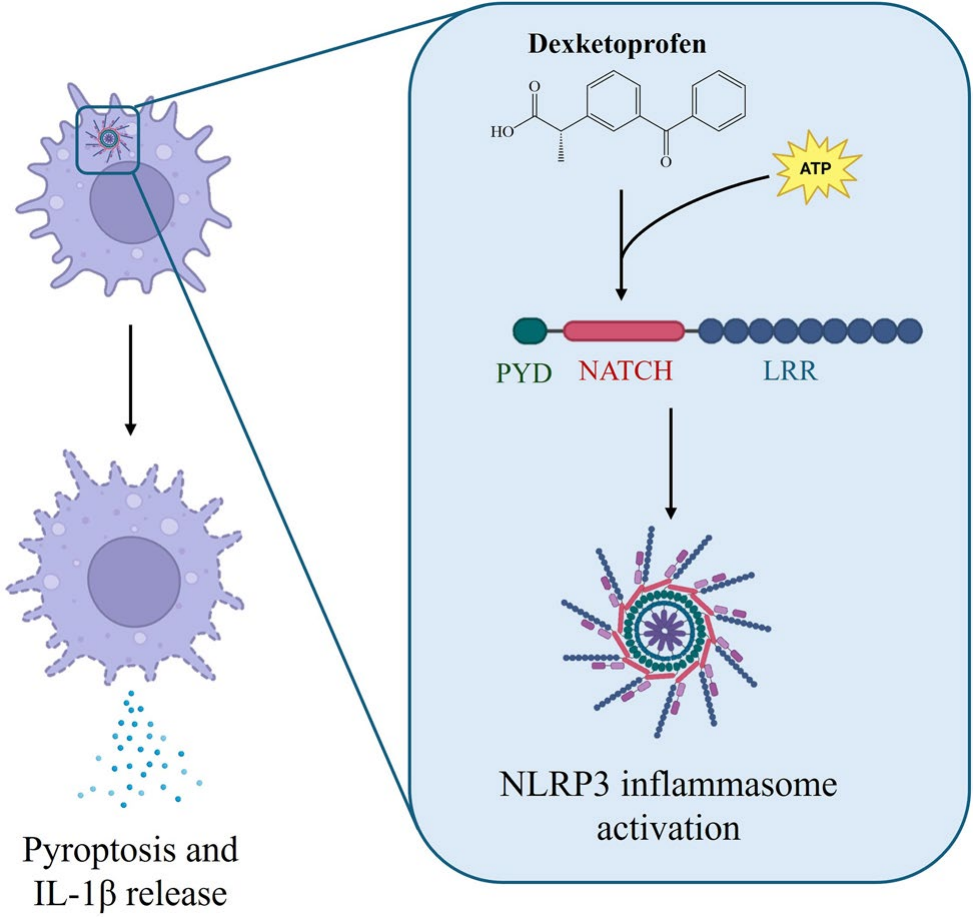

**Supplementary Information:**

The online version contains supplementary material available at 10.1007/s10787-025-01928-2.

## Introduction

Inflammasomes are macromolecular structures whose activation comprises the oligomerization of several intracellular proteins. The formation of this platform involves danger signal recognition by the intracellular sensor, which recruits caspase-1, via the apoptosis-associated speck-like protein containing a caspase recruitment domain (ASC). Caspase-1 cleaves IL-1 family cytokines into their active forms, as well as gasdermin D, which triggers the release of the lytic N-terminus and its assembly within the plasma membrane, forming a pore through which pyroptosis occurs (Holley et al. 2024; Wang et al. 2021). Thus, inflammasome assembly leads to caspase-1 dependent cell death pathway, taking part of the innate immunity and ensuring homeostasis (Huang et al. 2021).

The NOD-like receptor protein 3 (NLRP3) stands out as the inflammasome that has been best characterized to date which is activated by diverse stimuli, for instance, lysosomal damage, mitochondrial dysfunction or altered ion exchange among other events (Paik et al. 2021; Akbal et al. 2022), but has also been associated with many inflammatory and immune-related diseases (Chen et al. 2023). In this respect, NLRP3 has been directly or indirectly associated with cryopyrin-associated periodic syndrome (CAPS) (Putnam et al. 2024; Molina-López et al. 2024), neurodegenerative pathologies (Holbrook et al. 2021) including Alzheimer’s disease (AD) (Zhang et al. 2023; Yao et al. 2023; Terzioglu and Young-Pearse 2023) or Parkinson’s disease (PD) (Huang et al. 2023; Han and Le 2023), and cardiometabolic disorders or hypertension (Cho et al. 2023; De Miguel et al. 2021), among others.

Considering the aforementioned, NLRP3 is clearly implicated in a myriad of diseases. Therefore, research has considered this complex as a promising therapeutic target. Several drugs have been proposed as potential inhibitors of this inflammasome (Shao et al. 2015), with MCC950 being the most widely reported NLRP3 inhibitor. It has been further implemented in animal models to elucidate the role of NLRP3in disease; unfortunately, clinical trials observed hepatoxicity (Li et al. 2022). Some strategies advocate blocking the activation of the complex such as MCC950, while others such as anakinra or canakinumab seek to stop the inflammatory cascade resulting from activation of this platform (Mangan et al. 2018). During the last years, several compounds have been described to inhibit NLRP3 inflammasome complex activation by a non-specific mechanism. However, although these compounds do not specifically address NLRP3, their suppressive activity on this complex offers a very appealing therapeutic approach. Within this line, dexketoprofen (DXK) or racemic ketoprofen, another type of NSAID, is noteworthy for its analgesic, antipyretic and anti-inflammatory properties (Moore and Barden 2008). In fact, it is commonly prescribed to treat mild to acute pain and is among the most consumed nonsteroidal anti-inflammatory drugs worldwide at all age ranges. However, DXK induces drug hypersensitivity reactions (DHRs) and has been included among the potentially inappropriate medications for prescription (Kuczyńska et al. 2022; Salas-Casinello et al. 2023; Puig et al. 2024). So, despite its widespread use, nothing is known about its effect on NLRP3 inflammasome. Consequently, re-assessing its activity would certainly be of therapeutic significance, considering the involvement of NLRP3 in a large spectrum of pathologies.

## Results

### Dexketoprofen increases NLRP3 inflammasome response to different stimulus

**Fig. 1:**
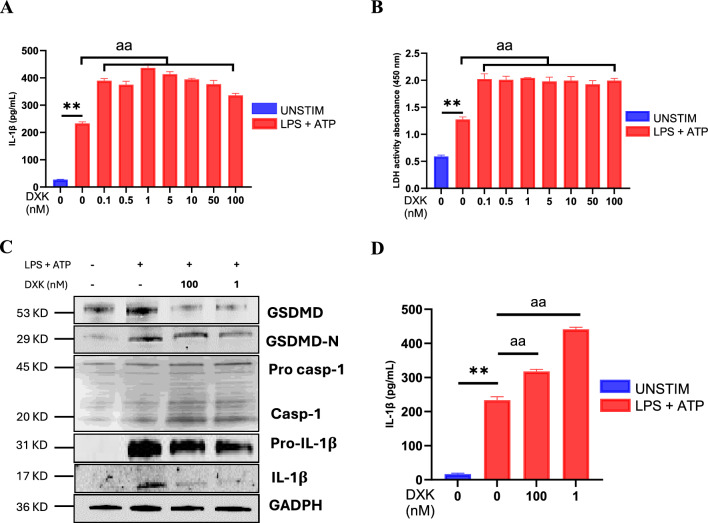
DXK effect in human macrophages when stimulated with LPS and ATP. **A** Supernatant IL-1β concentration measured by ELISA from macrophage unstimulated and primed with LPS 1 μg/mL for 4 h, stimulated with ATP 5 mM for 30 min and treated with different concentrations of DXK. **B** LDH activity in the supernatant. **C** Images of western blot analysis of macrophages stimulated with LPS and ATP and treated with DXK 100 nM and 1 nM. **D** IL-1β release in supernatant measured by ELISA. Data are mean + SEM from *n* = 3 independent experiments. Data were analyzed using a Student’s *t* test. ns = *p* > 0.05, *,^a^ = *p* ≤ 0.05, **,^aa^ = *p* ≤ 0.01. * represents LPS-ATP vs control and ^a^ represents DXK treatment vs LPS-ATP

To study the effect of DXK on the human macrophage inflammasome pathway, in the first assay, macrophages were treated with a wide range of concentrations of DXK to stimulate inflammasome activation. For this, macrophages were primed with 1 ng/mL LPS for 4 h and then stimulated with 5 mM ATP for 30 min. We observed an increase in both IL-1β release and cell death, showed by increased LDH levels, in macrophages when treated with DXK and activated with LPS and ATP (Fig. 1A, B). Statistical differences were observed from the lowest concentration of DXK (0.1 nM) to the highest (100 nM), which indicates that DXK can promote inflammasome activation even at very low concentration.

Next, we tested DXK at 1 nM and 100 nM to analyze by western blot whether DXK affects protein expression in human macrophages when activated by LPS and ATP. We detected a strong increase in caspase-1 expression and gasdermin D, showing a high activation of the NLRP3-inflammasome; however, we noticed a lower amount of IL-1β when cells are treated with DXK (Fig. 1C). A supernatants analyzed by ELISA showed that macrophages treated with DXK, LPS and ATP release a higher amount of IL-1β compared to LPS and ATP only (Fig. 1D). These evidences lead us to think that DXK might induce NLRP3 inflammasome activation and accelerate the release of IL-1β, increasing inflammation in the presence of an inflammatory stimulus such as LPS and ATP.

**Fig. 2:**
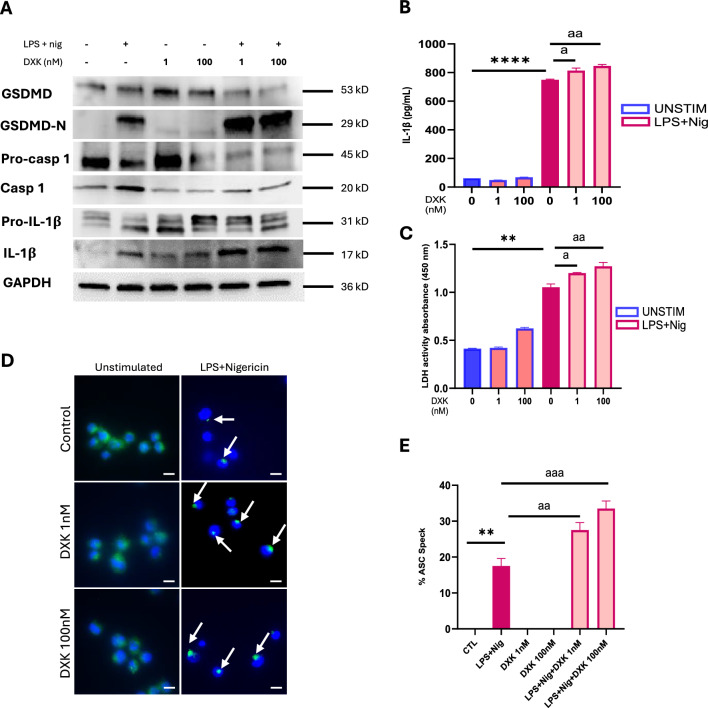
Effect of DXK alone and with inflammatory stimulation in human macrophages. **A** Representative images of western blot analysis of macrophages exposed to DXK 100 nM for 15 min and 1 nM, primed with LPS 1 μg/mL for 4 h stimulated with nigericin 10 µM for 30 min. **B** IL-1β concentration in the supernatant measured by ELISA. **C** LDH activity measured in the supernatant. **D** DeltaVision confocal images of human macrophages exposed to DXK 100 nM and 1 nM, primed with LPS and stimulated with nigericin. Macrophages were labeled with ASC (green) and DAPI (blue). Scale bars, 10 µm. Data are mean + SEM from *n* = 3 independent experiments. **E** The percentage of ASC speck-containing cells was quantified using the imaging software Fiji. For each individual experiment, the average number of ASC specks per nucleus was calculated and plotted. A total of 100 cells per condition were imaged and quantified. Data are mean + SEM from *n* = 3 independent experiments. Data were analyzed using a Student’s *t* test. ns = *p* > 0.05, *,^a^ = *p* ≤ 0.05, **,^aa^ = *p* ≤ 0.01. * represents treatment vs control and ^a^ represents treatment vs LPS-Nig

To study whether DXK enhances NLRP3 inflammasome activation after ATP independent stimulus, macrophages were stimulated with LPS and nigericin as inflammatory stimulus with and without DXK. Western blot analysis reveals that DXK alone cannot induce a well-defined inflammation compared to control, far from the toxicity of a drug with these characteristics. Expression of pro-inflammatory proteins such as caspase-1, GSDMD N-terminal and IL-1β were not changed with DXK concentration (Fig. 2A). However, DXK also enhances inflammation in macrophages when exposed to a non-ATP inflammatory stimulus such as LPS and nigericin. GSDMD N-terminal and IL-1β expression are higher when exposed to DXK to both concentrations and stimulated with LPS and nigericin (Fig. 2A). Furthermore, there was no statistical difference of IL-1β concentration in extracellular medium between macrophages exposed to DXK and control (Fig. 2B) corroborating that DXK alone has no effect in IL-1β release but IL-1β concentration in supernatant were increased when DXK was accompanied with LPS and nigericin compared to only stimulated (Fig. 2B). Furthermore, the difference of IL-1β releases compared with non-stimulated after ATP was significantly more increased than after nigericin stimulus and showed a more fast release after ATP than nigericin. Next, we analyze cell death by LDH activity in the supernatant. Cell death increases when stimulated with LPS and nigericin compared to the control. We also noticed an increase in cell death when macrophages were exposed to DXK 1 nM and 100 nM and stimulated with LPS and nigericin compared to cells stimulated alone (Fig. 2C). Here, Nlrp3 deficiency completely ablated DXK-induced IL-1β secretion (Supplementary Fig. 1).

We wanted to find out more about the effect of DXK by analyzing ASC specking on macrophages exposed to DXK and stimulus. We did not observe increased ASC specking on macrophages exposed to DXK compared to control (Fig. 2D, E), suggesting that DXK alone cannot induce inflammation on human macrophages. However, macrophages stimulated with LPS and nigericin exposed to DXK showed a higher increase in ASC specking compared to cells only stimulated and curiously with increased size observed in the immunofluorescence images.

### DXK facilitates ATP hydrolysis by NLRP3

**Fig. 3:**
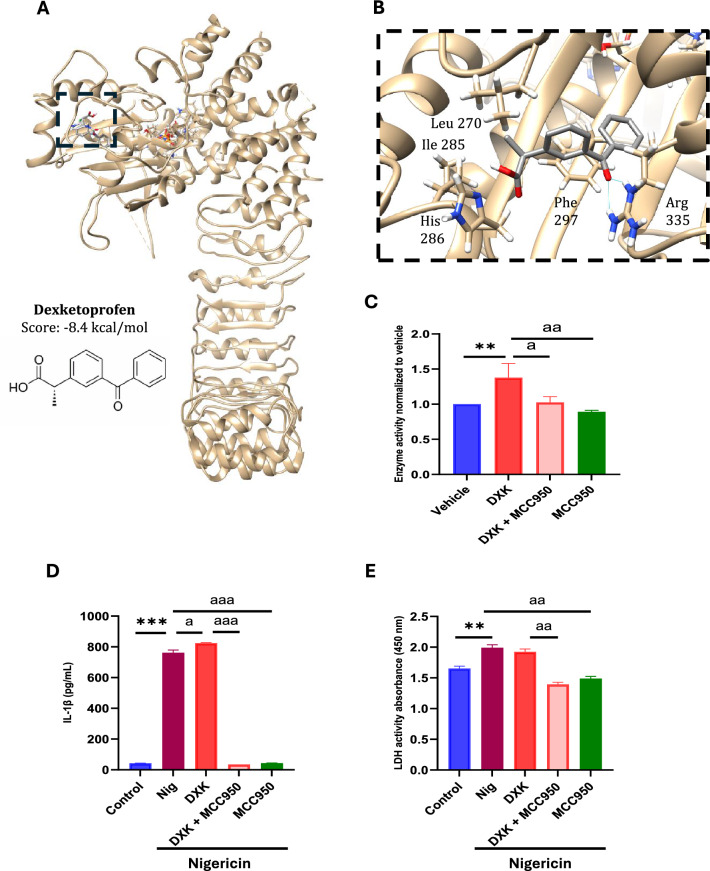
Combined effect of DXK and MCC950 in human macrophages when exposed to nigericin. **A** DXK docked to the NATCH domain of human NLRP3 (PDB:6NPY) using *AutoDock Vina*. **B** Chemical structure of DXK and zoom-in of the binding pocket with a score of -8.4 kcal/mol. Hydrogen bonds in blue. **C** ATPase activity measured by ADP-Glo Kinase Assay Kit using purified human NLRP3 exposed 15 min to vehicle, DXK 10 µM, MCC950 10 µM and both before adding purified ATP 250 µM. Data of enzyme activity were normalized to vehicle. **D** IL-1β concentration in the supernatant from macrophages exposed 15 min to DXK 100 nM, MCC950 10 µM and both before nigericin 10 µM 45 min. **E** LDH activity measured in the supernatant. Data are mean + SEM from *n* = 3 independent experiments. Data were analyzed using a Student’s *t* test. ns = *p* > 0.05, *,^a^ = *p* ≤ 0.05, **,^aa^ = *p* ≤ 0.01. * represents treatment vs control and ^a^ represents treatment vs Nig in **C** and D. * represents DXK vs control and ^a^ represents MCC950 vs DXK and vs Nig in **E**

Our findings about the difference between IL-1β release after ATP or nigericin show that DXK induces a more accelerated activation of inflammasome in an ATP-dependent induction. To understand how DXK enhances inflammation when exposed to an inflammatory stimulus, it is important to know where DXK binds; therefore, we ran a docking analysis using the NLRP3 protein (PDB:6NPY) and observed that DXK binds to the NATCH domain near the ATP binding pocket (Fig. 3A). DXK seems to have electrostatic interactions between the Ile285 and methylene group at 2.60 Å, and Leu270 at 2.85 Å and His286 at 2.48 Å with carboxylic acid group. We also noticed a π–π interaction between Phe297 and one phenyl group of DKS with a distance of 3.68 Å and two hydrogen bonds between two amine groups of Arg355 and the oxygen of ketone group of DXK at a distance of 2.01 Å and 2.27 Å. DXK binds to these residues with a score of − 8.4 kcal/mol (Fig. 3B).

Bearing in mind that DXK accentuates the production of pro-inflammatory cytokines in human macrophages when exposed to an inflammatory ATP stimulus, docking analysis findings show that DXK binds NLRP3 NATCH domain inducing a conformational change that opens the ATP pocket, facilitating its entrance and hydrolysis. To validate the effect of DXK as an NLRP3 ATPase activity enhancer, we ran an ATPase assay using purified human NLRP3 protein exposed to DXK and analyzing ADP generated. Indeed, because MCC950 has been described to bind NLRP3 ATPase activity resulting in the suppression of NLRP3 inflammasome assembly and activation, we include MCC950 treatment to study if this could neutralize the effect of DXK. DXK and MCC950 were tested at 10 µM in a solution of purified human NLRP3 1 µg/mL in kinase buffer. We observed an increment of NLPR3 ATPase activity when treated with DXK compared to vehicle (Fig. 3C). When purified, NLRP3 is exposed to MCC950 with and without DXK, and a lower ATPase activity is noted compared to DXK treatment. This data shows that DXK enhances human NLRP3 ATPase activity; nevertheless, MCC950 neutralizes DXK effect. To corroborate the NLRP3 specificity of DXK to induce activation, macrophages were primed with LPS and transfected with flagellin or poly dA-dT, using Lipofectamine and DXK to evaluate NLRC4 or AIM2 activation, respectively. Neither NLRC4 nor AIM2 inflammasome-dependent IL-1β release was influenced by DXK (Supplementary Fig. 2A and B).

Afterward, we wanted to evaluate the effect of nigericin in human macrophages when treated simultaneously with DXK and MCC950 as NLRP3 ATPase activity inhibitor (Coll et al. 2019). For this, we exposed macrophages to DXK 100 nM, MCC950 10 µM and both for 15 min before adding nigericin 10 µM for 45 min. The supernatants were collected and the IL-1β concentration was analyzed by ELISA and cell death by LDH activity.

IL-1β release ELISA shows that DXK in the presence of nigericin elevates the amount of IL-1β released by macrophages compared to nigericin alone (Fig. 3D). Nevertheless, macrophages stimulated with nigericin and exposed to MCC950 reduce significantly the IL-1β concentration even in the presence of DXK in the supernatant compared to those only stimulated.

We observed differences in cell death (Fig. 3E) when treated with nigericin and nigericin plus DXK compared to control without treatment. We also noticed differences in cells when exposed to nigericin and MCC950 with and without DXK compared to nigericin alone.

## Discussion

NLRP3 is one of the most studied and described inflammasome allowing us to perform a docking analysis revealing that DXK binds to the NLRP3 NATCH domain. The DXK binding site is near Ser295, the phosphorylation of which induces NLRP3 activation (Sandall and MacDonald 2019). We hypothesized that DXK would induce a conformational change that facilitates ATP entrance to its binding pocket, enhancing ATP hydrolyzation to phosphorylate Ser295 and promoting NLRP3 activation. We validated this hypothesis by an ATPase assay on purified human NLRP3. DXK increases NLRP3 ATPase activity, while double the presence of an NLRP3 ATPase activity inhibitor like MCC950 neutralizes the effect of DXK (Coll et al. 2019). We validated this DXK and MCC950 double treatment in vitro stimulating macrophages with nigericin. MCC950 lowers the pro-inflammatory effect of nigericin, reducing IL-1β release and cell death, while DXK increases IL-1β release without increasinging cell death. However, combined treatment of DXK and MCC950 reduces both IL-1β release and cell death, meaning that MCC950 diminishes inflammation produced by nigericin even in presence of DXK.

Considering that NLRP3 is related to several autoinflammatory disorders like cryopyrin-associated periodic syndrome (CAPS), Alzheimer (AD) and Parkinson (PD) diseases, diabetes, atherosclerosis and cardiovascular diseases, it is important to mind that DXK is a widely consumed NSAID now that we proved that DXK enhances inflammation in human macrophages. While NSAIDs have been shown described to inhibit inflammasome, this is not completely true given that other studies showed that ibuprofen failed to inhibit the NLRP3 inflammasome complex (Daniels et al. 2016); however, for example, on the other hand, ibuprofen has been shown to induce NLRP3 activation and pyroptosis in anaplastic thyroid cancer cells, which was contrary to the common hypothesis (Guo et al. 2024). Indomethacin, another NSAID, has also been shown to induce inflammasome activation in intestinal mucosa provoking enteropathy (Higashimori et al. 2016). Our findings concur with the previous studies proposing that depending on the treatment context, NSAIDs could be anti- or pro-inflammatory associated with inflammasomes.

Also, there have been studies suggesting that high doses of NSAIDs for long periods of time increase cardiovascular and cerebrovascular risk (McGettigan and Henry 2011) due to their mechanism as COX2 inhibitor (Abraham et al. 2007). Recent studies advise that the greater the inhibition of COX-2 and the lesser the inhibition of COX-1, the higher the thrombotic risk associated with NSAIDs (Grosser et al. 2010), suggesting that selective COX2 inhibition as DXK may be of risk, especially when used at high doses for an extended period of time. That is why we should aim to use NSAIDs at the lowest effective dose and for the shortest duration possible.

This study focuses on the effect of DXK in NLRP3-dependent inflammation pathway in human macrophages, which have more elevated expression of proteins and sensors related to inflammation, such as NLRP3, than other cell lines (Awad et al. 2017). It would be of great interest to study the global effect on different cell lines and in vivo models to determine whether this side effect of DXK could be risky in long-term treatment. Lately, MCC950 has been reported to improve cognitive function in AD rat model by inhibiting NLRP3-dependent neuroinflammation (Naeem et al. 2024), suggesting that MCC950 and DXK double treatment might be of great interest to study despite DXK’s side effects.

This study identifies DXK as an inflammatory inductor in human macrophages. We tested a wide range of concentrations of DXK and observed that even at low doses, there is a significant an increase of IL-1β release and cell death when exposed to DXK and a pro-inflammatory stimulus compared to those only stimulated. Induction of inflammation in macrophages with LPS and ATP results in inflammasome activation and release of caspase-1, which trigger the release of IL-1β and N-terminal gasdermin D fragment, leading to inflammation and cell death by pyroptosis. We observed that DXK exposure to stimulated human macrophages raises the amount of caspase-1 inside the cell, resulting in an increase and accelerated IL-1β release. In this respect, rapid secretion of IL-1β has been previously proposed with reduction of the intracellular active form, which probably would be a mechanism to control the inflammation level (MacKenzie et al. 2001; Semino et al. 2018). We also noticed that IL-1β expression in cells is lower in the presence of DXK and on stimulation with LPS and ATP compared to macrophages that were only stimulated. Therefore, we observed an increase in IL-1β release when exposed to DSK, which leads us to think that most IL-1β produced by macrophages were already released into the extracellular medium when cells and supernatant were collected. We observed that macrophages stimulated with LPS and ATP and exposed to low doses of DXK releases more IL-1β than those exposed to high doses. Conceivably, doses of DXK lower than 100 nM combined with a pro-inflammatory stimulus such as LPS and ATP might reduce pyroptosis, since we observed lower expression of N-terminal gasdermin D fragment. Consequently, macrophages exposed to low doses of DXK would live longer than those exposed to high doses of DXK, releasing a greater amount of IL-1β to the extracellular medium.

## Limitations

Despite the relevance and novelty of our results, we recognize some limitations of our study. First, we worked with macrophages, but to study the in vivo implications it would be very informative to include human samples from patients documented to show adverse effects or hypersensitivity. Second, an in vivo animal model would be recommended to corroborate our findings and also to evaluate the specific tissues associated with the DXK-dependent NLRP3 activation.

Considering these facts, DXK has proven to induce a slight inflammation when exposed alone to macrophages and enhances inflammation in the presence of different pro-inflammatory stimuli. For this reason, we strongly suggest further in vitro and in vivo studies to evaluate continued treatment with DXK, because it has been reported that prolonged use of NSAIDs such as celecoxib, naproxen or ibuprofen is associated with cardiovascular risk (Nissen et al. 2016).

## Material and methods

### Reactants

Phorbol 12-myristate-13-acetate (PMA) (P8139-MG, Sigma Chemical Co., St. Louis, MO, USA), dexketoprofen (sc-357330, Santa Cruz Biotechnology, Santa Cruz, CA, USA), lipopolysaccharide (LPS) (L4391, Sigma Chemical Co., St. Louis, MO, USA), nigericin (sc-201518B, Santa Cruz, CA, USA), adenosine triphosphate (ATP) (sc-202040A, Santa Cruz, CA, USA), bovine serum albumin (BSA) (9048–46-8, Sigma Chemical Co., St. Louis, MO, USA), DAPI (Santa Cruz Biotechnology, Santa Cruz, CA, USA), saponin (47,036, Sigma Chemical Co., St. Louis, MO, USA), bioBLUPrestainesProtein loader (GTPBM0003, Biorad Laboratories Inc., Hercules, CA, USA). Primary antibodies: gasdermin D (ab219800, Abcam, Cambridge, UK), IL-1β (ab243091, Abcam, Cambridge, UK), caspase-1 (NBP1-45,433, Novus Biologicals, Colorado, USA), GADPH (5174S, Cell Signaling, Danvers, MA, USA), NLRP3 (NBP2-12,446, Novus Biologicals, Colorado, USA) and ASC (13833S, Cell Signaling, Danvers, MA, USA). Secondary antibodies: rabbit (401,353-2ML, Burlington, MA, USA) and mouse (401,253-2ML, MilliporeSigma, Burlington, MA, USA).

### Cell culture

Cells were incubated at 37 °C in a 5% CO_2_ atmosphere. Primary human THP1 monocytes (TIB-202, ATCC, Manassas, VA, USA) were cultured in RPMI-1640 medium (Gibco, Invitrogen, Eugene, OR, USA) supplemented with 10% fetal bovine serum (FBS) (Gibco, Invitrogen, Eugene, OR, USA) and 1% antibiotics solution (L0010-100, Nuaillé, France). Macrophages were obtained from THP1 cells differentiation, PMA 50 ng/mL 24 h.

Macrophage cells were treated with dexketoprofen (1 nM or 100 nM for 15 min) and then primed with LPS 1 µg/µL 4 h and stimulated with ATP 5 mM or nigericin 10 µM for 30 min. Cells were scrapped and centrifuged for 5 min at 1200*g*. Supernatant and pellet was stored at – 20 °C for further assays.

### Protein extraction

Pellet cells were lysed using RIPA (Thermo Fisher Scientific, MA, USA) with PMSF (Thermo Fisher Scientific, MA, USA) 1 nM as lysis buffer. Cells were resuspended in lysis buffer and kept in ice for 30 min and centrifuged for 5 min at 12.000*g* and the supernatant was collected. Protein quantification was done using the BCA kit (Thermo Fisher Scientific, MA, USA). Then, protein aliquots 10 ng/µL were mixed with NuPAGE LDS sample buffer (Invitrogen Eugene, OR, USA) and subjected to thermal shock for 5 min at 95 °C.

### Electrophoresis

Samples were loaded in mini-PROTEAN TGX precast gels 4–20% (4,561,096, Biorad Laboratories Inc., Hercules, CA, USA) and ran in Tris/glycine/SDS buffer (1,610,772, Biorad Laboratories Inc., Hercules, CA, USA) 45′ 200 V.

### Inmunoblot

Proteins were transferred from gel 0.45 µm (1,620,115, Biorad Laboratories Inc., Hercules, CA, USA) using a TransBlot Turbo (Biorad Laboratories Inc., Hercules, CA, USA). Membranes were blocked by immersion in a BSA 5% solution during 1 h with gentle agitation. Primary antibodies 1:1.000 were incubated overnight at 4 °C. Membranes were washed three times with PBS–Tween20 solution and incubated with secondary antibody 1:10.000 for 1 h at room temperature. Membranes were washed three times and developed in a ChemiDoc MP Imaging System (Biorad Laboratories Inc., Hercules, CA, USA) using WesternBright Sirius detection kit (Advansta, San Jose, CA, USA).

### Lactate dehydrogenase (LDH) activity assay

LDH activity was measured using the LDH Assay Kit/Lactate Dehydrogenase Assay Kit (Colorimetric) (ab102526, Abcam, Cambridge, UK) following the recommended procedure. In a 96-well plate, supernatant samples and reaction mix were added. The plate was analyzed every 2–3 min for at least 30 min with iMark microplate reader (Biorad Laboratories Inc., Hercules, CA, USA) in kinetic mode at 37 °C.

### ELISA (enzyme-linked immunosorbent assay)

Supernatant IL-1β levels were analyzed using human IL-1 beta ELISA Kit (ab214025, Abcam, Cambridge, UK) and following the recommended procedure. Samples and antibody cocktail were added to each well and incubated for 2 h at room temperature. Each well was washed three times and the development solution was added. After 10 min incubation in the dark, stop solution was added and OD at 450 nm was record using an iMark microplate reader.

### Immunofluorescence assay

Macrophages were grown on 1 mm-width glass coverslips in RPMI-1640 medium containing 10% FBS and 1% antibiotics. After inflammasome treatment, cells were washed twice with PBS, fixed in 10% paraformaldehyde for 10 min at room temperature, washed three times with PBS and permeabilized with 0.1% saponin in PBS for 30 min at room temperature and incubated in blocking buffer (BSA 1% in PBS) for 1 h. After blocking, cells were incubated overnight at 4 °C with a 1:100 primary antibody solution in blocking buffer. Cells were washed three times with PBS and incubated with a 1 µg/ml DAPI solution in PBS for 5 min and washed five times with PBS. Glass coverslips were mounted on microscope slides using Vectashield Mounting Medium (Vector Laboratories, Burlingame, CA, USA) and visualized using a DeltaVision microscope (imsol, Preston, UK).

### Docking study

Dexketoprofen mol2 and human NLRP3 pdb file and file were used for molecule–protein interaction study. Dexketoprofen mol2 structure file was designed using GaussView 5.08 software. Atoms’ distance, angles and polar charges were optimized using Gaussian09W software. Human NLRP3 pdb file was obtained as 6NPY file from RCSB Protein Data Bank. NEK7 structure was removed from 6NPY file using UCSF Chimera 1.16 software before docking analysis. Polar hydrogens and Gasteiger charges were added using UCSF Chimera 1.16 software. Autodock Vina software was used for docking validation.

For human NLRP3 (PDB:6NPY), a grid box was made with x, y, z dimensions of 37 Å, 32 Å and 36 Å centered with x, y, z coordinates of 93.177 Å, 96.763 Å and 81.168 Å. Non-polar hydrogens and lone pairs were removed, water molecules and chains of non-standard residues were ignored and all non-standard residues were not ignored for the human NLRP3 (PDB:6NPY) receptor. Non-polar hydrogens and lone pairs were not removed for the dexketoprofen Ligand. Docking analysis was done based on maximum exhaustiveness of search and maximum energy difference of 2 kcal/mol. Ten binding modes were given after analysis which were visualized and analyzed using UCSF Chimera 1.16 software. Interaction type and distance between dexketoprofen atoms and residues were analyzed using structure measurements tool provided by UCSF Chimera 1.16 software.

### ATPase assay

Purified recombinant human NLRP3 (Novus Biologicals) was incubated at 37 °C with dexketoprofen and MCC950 10 mM for 15 min in the reaction buffer. ATP (250 μM, Ultra-Pure ATP) was then added and the mixture was further incubated at 37 °C for another 40 min. The amount of ATP converted into adenosine diphosphate (ADP) was determined by luminescent ADP detection with ADP-Glo Kinase Assay Kit (Promega, Madison, MI, USA) according to the manufacturer’s protocol. The results were expressed normalized to the enzyme activity of NLRP3 treated with vehicle.

Luminescence was measured using a Typhoon FLA 9500 Biomolecular Imager (Molecular Dynamics, Sunnyvale, CA, USA) and data were processed using ImageQuant™ software.

### Statistics

All data are expressed as means ± SEM. Statistical differences among the different groups were measured using an unpaired Student’s *t* test. A *P* value of ≤ 0.05 was considered statistically significant. Statistical analyses were performed using Prism software version 5.0a (GraphPad, San Diego, CA). Asterisks in the figures represent the following: * *P* ≤ 0.05; ** *P* ≤ 0.01; and *** *P* ≤ 0.001.

## Supplementary Information

Below is the link to the electronic supplementary material.

Supplementary file1 (PDF 171 KB)

## Data Availability

The data that support the findings of this study are available from the corresponding author upon reasonable request.
