# Supplementary material for: Dexketoprofen enhances NLRP3 activation via ATPase activity after canonical stimuli
Source: Inflammopharmacology. 2025 Sep 23;33(10):6089–98. doi: 10.1007/s10787-025-01928-2 (PMC12552314; doi:10.1007/s10787-025-01928-2)
Supplement: Supplementary file 1 — Supplementary file1 (PDF 171 KB) [file 10787_2025_1928_MOESM1_ESM.pdf]

Dexketoprofen enhances NLRP3 activation via ATPase activity after canonical stimuli

Daniel Boy-Ruiz[1], Juan Miguel Suarez-Rivero[1], Inés Muela-Zarzuela[1], Mario D. Cordero [1]

[1] Department of Molecular Biology and Biochemical Engineering, Universidad Pablo de Olavide, 41013 Seville, Spain.

Running Title: Dexketoprofen activates NLRP3-Inflammasome complex

Corresponding Author:

Dr. Mario D. Cordero

Department of Molecular Biology and Biochemical Engineering, Universidad Pablo de Olavide, 41013 Seville, Spain. Email: mdcormor1@upo.es.

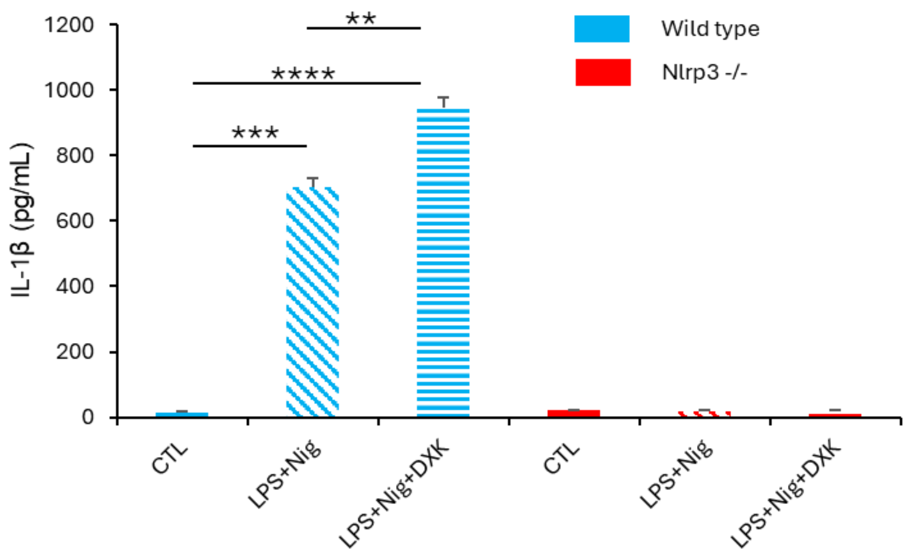

Supplementary Figure 1. NLRP3 deletion suppressed DXK-dependent activation associated with Nigericin. Supernatant IL-1β concentration measured by ELISA from macrophages unstimulated and primed with LPS, stimulated with nigericin and treated with DXK. Data were analyzed using a Student's *t*-test. *** $p > 0.01$, **** $p \leq 0.001$.

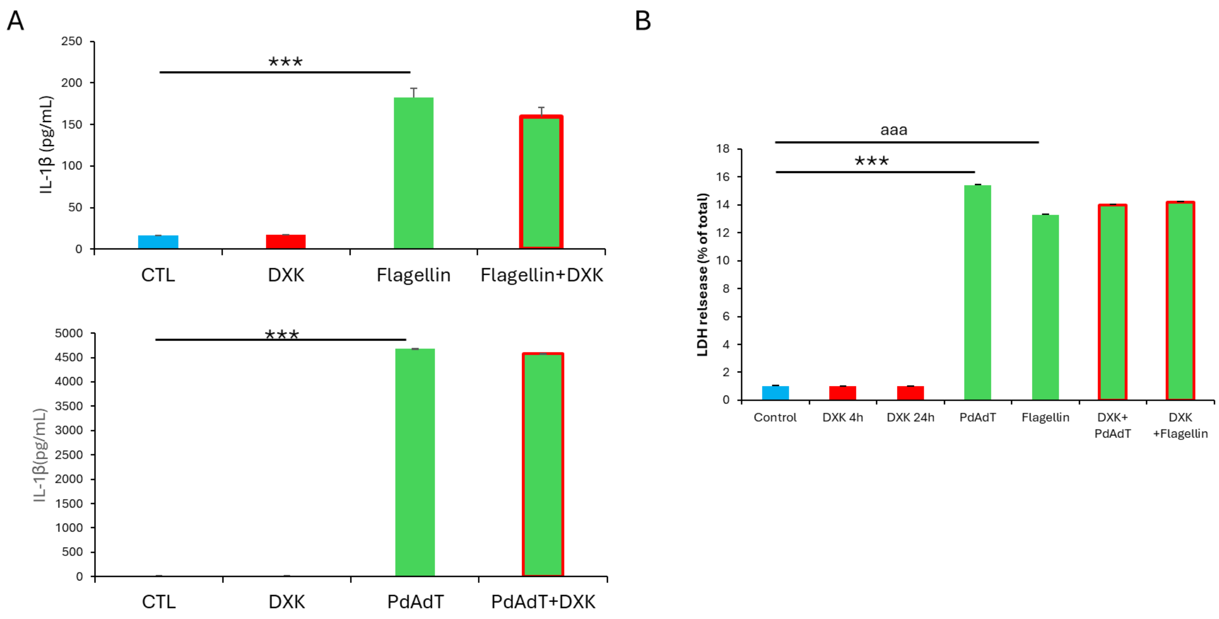

Supplementary Figure 2. NLRC4 and AIM2 are not stimulated by DXK. **A.** Supernatant IL-1β concentration measured by ELISA from macrophages unstimulated and primed with LPS, stimulated with nigericin and treated with DXK. B. LDH activity in supernatant. Data were analyzed using a Student's *t*-test. *** and ^{aaa} $p > 0.001$.
